# Effect of S-ketamine on postoperative pain sensitivity in children with preoperative chemotherapy

**DOI:** 10.1038/s41390-025-04146-2

**Published:** 2025-05-30

**Authors:** Siyi Zhou, Yong Bian, Kan Zhang, Yue Huang, Jijian Zheng

**Affiliations:** Department of Anesthesiology, Shanghai Children’s Medical Center Affiliated to Shanghai Jiaotong University School of Medicine, Shanghai, 200127 China

## Abstract

**Background:**

Children undergoing chemotherapy experience exacerbated postoperative pain and prolonged pain perception. Intraoperative intravenous administration of S-ketamine can alleviate postoperative pain. However, its efficacy in mitigating chemotherapy-induced hyperalgesia remains uncertain. This study evaluates the effect of S-ketamine on postoperative pain sensitivity in children who received preoperative chemotherapy.

**Methods:**

A total of 40 children undergoing preoperative chemotherapy and scheduled for open abdominal surgery were recruited from our center and randomly assigned to either the S-ketamine group or the control group. The primary outcomes included postoperative mechanical pain threshold, FLACC scale, Wong-Baker FACES pain rating scale (WBS), and cases of additional analgesic use. Secondary outcomes included intraoperative hemodynamic changes, extubation time, and incidence of adverse events.

**Results:**

Thirty-six children were included in the study. The two groups had no significant difference in preoperative mechanical pain thresholds (*P *= 0.585). Patients receiving S-ketamine had higher mechanical pain thresholds at 24 and 48 h post-surgery (both *P *< 0.001). Preoperative FLACC and WBS were 0 in both groups. Postoperative FLACC and WBS showed significant differences at various time points (all *P *< 0.05). There is a negative correlation between infusion time of S-ketamine and postoperative mechanical pain threshold at 24 h (*r *= −0.570, *P *= 0.014) and 48 h postoperatively (*r *= −0.643, *P *= 0.004) in the S-ketmanie group.

**Conclusion:**

Intravenous S-ketamine significantly increases postoperative mechanical pain threshold and reduces pain in patients who received neoadjuvant chemotherapy.

**Impact:**

- Children undergoing chemotherapy experience exacerbated postoperative pain and prolonged pain perception. Intraoperative intravenous administration of S-ketamine can alleviate postoperative pain. However, its efficacy in mitigating chemotherapy-induced hyperalgesia remains uncertain. This study fills the gap in this area.
- This study evaluates the effect of S-ketamine on postoperative pain sensitivity in children who received preoperative chemotherapy.
- S-ketamine’s NMDAR antagonism may partly reduce pain sensitivity, thus reversing the pain effect.
- The results of this study provide promising evidence for the potential benefits of S-ketamine in improving postoperative pain outcomes in this patient population.
- The infusion time of S-ketamine ranged from 30 to 655 min, suggesting that the beneficial effect can be achieved within this time frame.

## Introduction

With the advancement of imaging technology, more retroperitoneal malignant solid tumors are being diagnosed. As a result, a more significant number of children may require surgical treatment. Retroperitoneal malignancies are intended to invade essential great blood vessels and vital organs, including the abdominal aorta, superior vena cava, and bilateral renal artery and vein, increasing the surgical resection’s complexity. The extent of surgical resection of the primary lesion directly affects the child’s prognosis.^1^ So, in some cases, neoadjuvant chemotherapy is often indicated to reduce tumor volume when complete surgical removal is not feasible.^2^ However, it was reported that about 78% of pediatric patients using neurotoxic chemotherapy drugs would have chemotherapy-induced peripheral neurotoxicity (CIPN).^3^ CIPN can persist six months or even longer after the patient’s last chemotherapeutic treatment and can significantly impact the quality of life in children.^4^ Moderate-to-severe sensory abnormality is the most common adverse effect of CIPN, which is often caused by various neurotoxic drugs, with a prevalence of 2 ~ 40% in pediatric patients. Some severe manifestations of sensory abnormality include numbness, tingling, neuropathic pain, muscle weakness, and hyperalgesia.^5–8^ Previous studies have shown that patients with CIPN will suffer aggravated postoperative pain for a longer duration.^9,10^

No consistent, evidence-based preventative or therapeutic strategies for CIPN have been established in children.^5^ N-methyl-D-aspartate receptor (NMDAR) antagonist, is one of the primary drugs used to treat CIPN.^11,12^ S-ketamine, the S (+)-isomer of ketamine, provides twice the analgesic effect of racemic ketamine and possesses the advantages of a lower incidence of side effects like hallucinations and dissociation.^13^ Some studies have confirmed that S-ketamine can effectively improve the hyperalgesia induced by various reasons, including drug-induced hyperalgesia and peripheral neuropathies.^11,12,14,15^ Further research is needed to evaluate its efficacy and safety.

Low-dose ketamine has been successfully used in treating chronic pain characterized as nociceptive and neuropathic pain in patients with cancer.^16,17^ Theoretically, S-ketamine might also be promising in the perioperative pain management of patients with CIPN. There are few studies about the utility of S-ketamine in pediatric chemotherapy patients undergoing surgery, and the effect of S-ketamine on postoperative pain in these children is still unclear. It is necessary to conduct a study to ensure the safety and efficacy of treatment options of S-ketamine in these specific populations.

In this study, our objective was to explore the effect of S-ketamine on postoperative pain in children receiving neoadjuvant chemotherapy, and we observed whether the addition of a low dose of S-ketamine can relieve the postoperative pain in children receiving neoadjuvant chemotherapy and prevent the chronicity of acute pain.

## Methods

This prospective, randomized, controlled trial was approved by the Institution Review Board of Shanghai Children’s Medical Center (OSCMCIRB-K2020056-1) and was registered at the Chinese Clinical Trial Registry (ChiCTR2000033994). It was carried out over the period from July 2020 to February 2022.

### Participants

Eligible patients with malignant solid tumors, including neuroblastoma (NB), Wilms tumor (Wilms tumor), and hepatoblastoma (HB), aged between 3 and 12, ASA Physical Status I-Ш, and undergoing open abdominal surgery were recruited in this study. Exclusion criteria included the use of other analgesics within 48 h before the operation, patients’ body mass index (BMI) > 30 kg/m^2^, patients with complicated cardiovascular disease or other severe systemic diseases, patients not being treated for the first time with neoadjuvant chemotherapy, patients with a history of chronic pain before neoadjuvant chemotherapy, or any other conditions not suitable for the trail.

### Randomization

In our study, participants were randomly assigned to one of two groups: the S-ketamine group (Group S) or the control group (Group C) in a 1:1 ratio using a computer-generated randomization program in SAS software, version 9.4 (SAS Institute Inc., Cary, NC). The randomization was achieved by an independent research nurse who was not involved in recruitment, treatment administration, or outcome assessment. The research nurse prepared individual opaque, sealed envelopes containing computer-generated instructions for all participants to ensure allocation concealment further. The envelopes were only opened after confirming the eligibility of the participants and obtaining their consent. The researchers involved in implementing the study, the participants, and the perioperative care providers were all blinded to the group assignment throughout the entire period.

### Interventions

On the day of surgery, prior to the patient entering the operating room, an anesthesiologist who is not involved in the patient’s perioperative care opened the envelope, and participant allocation was assigned. Then, this anesthesiologist was responsible for preparing the investigational drug, labeling it with specified induction and maintenance dosages, and delivering the prepared medication to the attending anesthesiologist overseeing the respective surgical procedure. All patients fasted for 6 h, abstained from liquid for 2 h before surgery, and did not take any drugs. After the patient was positioned on the operating table, the ECG, pulse oximetry, and non-invasive blood pressure measurement monitors were placed, and then an intravenous line was established. Anesthetic induction was performed with midazolam 1 mg∙kg^−1^, sufentanil 0.2 μg∙kg^−1^, propofol 3 mg∙kg^−1^, rocuronium 0.6 mg∙kg^−1^, and atropine 0.1 mg∙kg^−1^. Mechanical ventilation was performed after tracheal intubation. The respiration parameters were as follows: oxygen/air mixed flow 2 L∙min^−1^, tidal volume 6 ~ 8 ml∙kg^−1^, inspiration: expiratory ratio 1:2, positive end-expiratory pressure (PEEP) 5cmH_2_O. The expiratory end-tidal CO_2_ was maintained between 35 ~ 45 mmHg during the whole surgery. Sufentanil was added 0.1 μg kg^−1^ before the start of surgery. Anesthesia was maintained with constant rate infusion of propofol 4 mg∙kg^−1^∙h^−1^, remifentanil 0.3 μg∙kg^−1^∙min^−1^ and rocuronium 0.6 mg∙kg^−1^∙h^−1^, with or without sevoflurane according to the hemodynamic changes during the surgery. 0.05 μg∙kg^−1^ of sufentanil was added before the surgeon sutured the skin incision. All patients received patient-controlled intravenous analgesia (PCIA) of continuous infusion (basal rate of 2 ml∙h^−1^) and a 1-ml on-demand bolus with 15 min of a lockout interval. PCIA included 2.5 μg∙ kg^−1^ of sufentanil and 0.2 mg∙kg^−1^ of tropisetron in a total volume of 100 ml and lasted for 48 h.

The children in the S-ketamine group were given 0.5 mg∙kg^−1^ of S-ketamine during the induction, maintained with S-ketamine 0.4 mg∙kg^−1^∙h^−1^ until 30 min before the end of the surgery. The control group was given the same volume of normal saline. The Electronic Von Frey Anesthesiometer (IITC Life Sciences, Woodland Hills, CA) was used to measure the patient’s mechanical pain threshold. The measure ranged up to 800 Gr. Measurement method: The patient was requested to close his eyes, and the investigator made the tip of the Frey hair contact with the skin within 5 cm around the operative incision. Pain threshold was measured as minimum pressure at the time when the patient reported a distinct change in pain perception. The mechanical pain thresholds of all patients were measured at three time points: before induction, 24 h after surgery, and 48 h after surgery. According to the above method, measurements were performed three times for each time, and the final result was taken the average of the measurement values.

The surgical time, intraoperative bleeding, blood transfusion volume, and hemodynamic changes were also recorded. Hemodynamic parameters included heart rate (HR) and mean blood pressure (MAP). All patients’ HR and MAP were collected at four time points: before induction (T0), at the time of intubation (T1), skin incision (T2), and extubation (T3). The investigator used the Face, Legs, Activity, Cry, Consolability (FLACC) scale to assess the degree of pain before induction, at the time of patients’ wake-up, 24 h after surgery, and 48 h after surgery. Meanwhile, self-reported pain levels were evaluated by the children themselves through the Wong-Baker FACES pain rating scale (WBS).^18,19^ The FLACC was divided into four degrees: painless <3 points: good; 3 ~ 4 points: basically satisfied; ≥6 points: poor; 10 points: severe pain. If the patient’s postoperative FLACC was >4, 0.05 μg∙kg^−1^ of sufentanil was given intravenously for analgesia in the post-anesthesia care unit (PACU), and 1 mg∙kg^−1^ of diclofenac potassium rectal suppository was given as a remedy when the patient returned to the ward.

The change of heart rate and blood pressure should not exceed 20% of the baseline. If the increase of HR or mean blood pressure MAP was more than 20% of the preoperative baseline, intravenous injection of 5 μg∙kg^−1^ of esmolol or 0.5 ~ 5 μg∙ kg^−1^ min^−1^ of nicardipine would be continuously administered, respectively. In contrast, if the decrease of heart rate or MAP was more than 20% of the preoperative baseline, 0.01 mg∙kg^−1^ of atropine intravenously or 0.01 ~ 0.05 μg∙kg^−1^∙min^−1^ of norepinephrine would be continuously administered after excluding the factors of anesthesia depth.

### Outcome measures

The characteristics of all children were collected, including age, sex, height, weight, diagnosis, and BMI. The primary outcome was the change in the mechanical pain threshold of children, the alterations of FLACC and WBS, and the number of cases requiring rescue analgesia. The secondary outcomes included the surgical time, intraoperative bleeding, blood transfusion volume, hemodynamic changes during the operation, the extubation time, and the incidence of adverse events.

### Sample size calculation

Based on previous studies, a difference in the FLACC scale of 3 points was considered to be clinically significant.^20^ Our pre-experiment found that the children undergoing malignant solid tumor surgeries had a median score of 3 of FLACC upon arrival at the PACU. Thus, A two-group Mann-Whitney test was used to detect a difference of 3 in the FLACC scale with α = 0.05 and 1-β = 0.8. A total sample size of 36 was calculated to be sufficient. Allowing for a dropout of 10% of subjects during the trial, we enrolled 40 patients in the study with a 1:1 ratio of 20 patients in each group.

### Statistical analysis

The statistical analyses were performed using the IBM SPSS Statistical 21.0 (IBM Corp., Armonk, NY) and GraphPad Prism 8.0.1 (GraphPad Inc.), and *P *< 0.05 was considered statistically significant. The chi-square test or Fisher exact test was used to compare categorical variables. The normally distributed outcomes measured were compared between groups using independent-sample t-tests. Non-normally distributed or ordinal results were compared using Wilcoxon rank sum tests.

Repeated measured data, including mechanical pain threshold, FLACC, Wong-Baker FACES pain rating scale, and hemodynamics results, were analyzed using generalized estimation equation models. An autoregressive correlation structure was used for clustered longitudinal data over time, and primary outcomes measured were compared at multiple time points with baseline if an interaction of group assignment and time was found.

## Result

### Patients’ characteristics

**Fig. 1:**
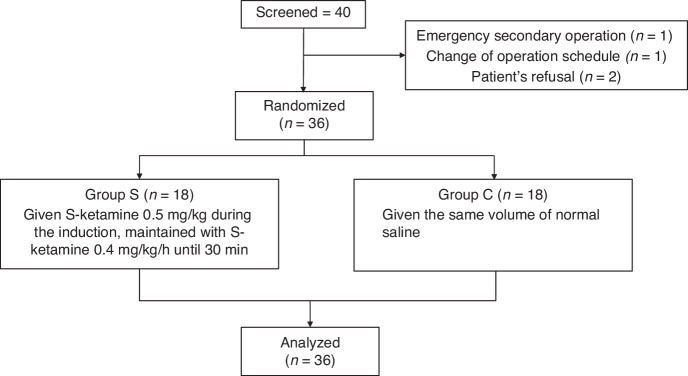
Patients flow diagram showing the numbers of individuals at each stage of the study.

From July 2020 to February 2022, 40 patients were enrolled in the study. Among four patients who failed to enter the study, one had to be withdrawn because of unscheduled emergency surgery afterward, one because of the cancellation of the surgery, and two other patients declined to enter the study after entering the operating theater. The patient flow is presented in Fig. 1. At last, 36 patients were included in the trial. The clinical characteristics of these patients and their operative conditions are shown in Table 1.

**Table 1:** Clinical characteristics of included children.

Characteristic	Group S (*n* = 18)	Group C (*n* = 18)	*P* value
Age, years	5.5 ± 2.6	5.1 ± 2.6	0.670
BMI, kg/m^2^	15.9 ± 1.7	16.1 ± 2.3	0.766
ASA (*n*, I/II/III)	1/7/10	2/7/9	0.930
Diagnosis (*n*, Wilms tumor/NB/HB)	2/13/3	4/13/1	0.252
Infused red blood cell volume, ml	213.9 ± 121.0	231.7 ± 129.0	0.686
Infused plasma volume, ml	61.1 ± 60.8	66.7 ± 68.6	0.799
Infused crystal volume, ml	1189.3 ± 424.6	1000.0 ± 337.8	0.161
Blood loss, ml	230.0 ± 112.9	252.2 ± 159.4	0.632
Surgical time, min	306.1 ± 175.3	253.7 ± 122.1	0.306
Gender (male/female)	9/9	11/7	0.526

Data are shown a as mean ± SD or numbers.

*BMI* Body Mass Index, *ASA* American Society of Anesthesiologists, *NB* neuroblastoma, *HB* hepatoblastoma.

### Mechanical pain threshold

**Fig. 2:**
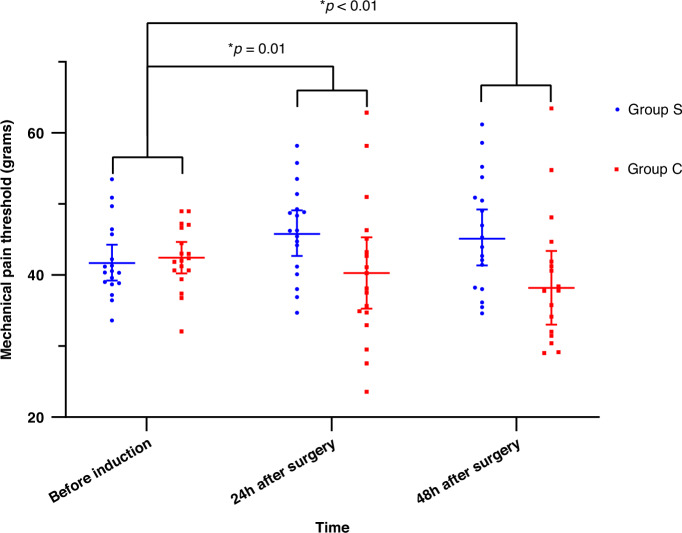
Mechanical pain threshold at different time points. The data are presented as mean ± SD, with individual data points shown as dots. A *p*-value < 0.05 was considered significantly different between the groups.

According to the GEE model, the difference in the mechanical pain thresholds between the two groups was statistically significant after adjusting for sex, age, Body Mass Index, and surgical duration (*P *= 0.033). The interaction effect between group assignment and time was also significant(*P *= 0.015). The mean differences of MPT at 24 and 48 h postoperatively differed significantly from the baseline mean differences. The results of GEE models are shown in Table 2, and the changes in the mechanical pain thresholds of each group at different time points are shown in Fig. 2.

**Table 2:** Mechanical pain threshold results according to the GEE model.

	All patients (*n* = 36)	Group S (*n* = 18)	Group C (*n* = 18)	$\triangle$MPT of different timepoints (95% CI)	Group *P* value	Time *P* value	Interaction *P* value
MPT(grams)					0.033	0.439	0.015
before induction	42.2 (40.7, 43.8)	42.0 (39.6, 44.3)	42.4 (40.4, 44.4)	Reference			
24 h after surgery	43.3 (40.6, 45.9)	46.2 (43.3, 49.1)	40.3 (35.8, 44.8)	6.4 (1.5, 11.2)^*^			0.01
48 h after surgery	42.0 (39.0, 45.0)	45.8 (42.2, 49.4)	38.2 (33.5, 42.9)	8.0 (2.3, 13.8)^*^			0.006

Data are shown as mean(95% CI).

*MPT* Mechanical pain threshold. $\Delta$*MPT* mean difference of MPT between two groups at 24 h after surgery or 48 h after surgery minus mean difference of MPT before induction between two groups. The covariates adjusted in these models included sex, age, BMI(Body Mass Index) and surgical duration.

^*^P < 0.05.

### FLACC, Wong-Baker FACES pain rating scale, and the number of cases requiring additional analgesics after surgery

**Fig. 3:**
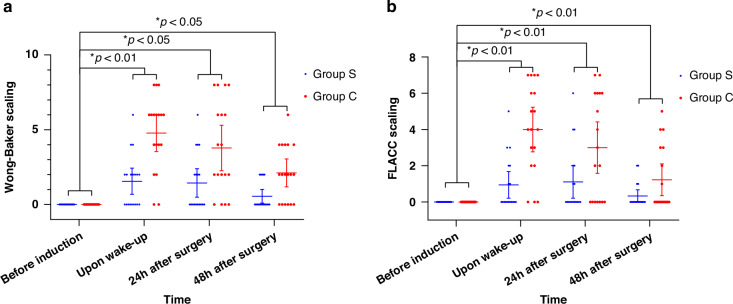
WBS and FLACC scale at different time points. **a** WBS at different time points and **b** FLACC scale at different time points. The data are presented as mean ± SD, with individual data points shown as dots. A *p*-value < 0.05 was considered significantly different between the groups.

Based on the GEE model results, the group assignment and time correlation were also found in the changes of FLACC and the Wong-Baker FACES pain rating scale (both *P* values were less than 0.001). Both mean differences differed significantly from the baseline mean differences in the results of FLACC and the Wong-Baker FACES pain rating scale at the time of awakening, 24 and 48 h postoperatively. The results of FLACC and the Wong-Baker FACES pain rating scale based on GEE models are shown in Table 3. The trend of FLACC and WBS is displayed in Fig. 3.

**Table 3:** Wong Baker faces and FLACC scaling results according to the GEE model.

	All patients (*n* = 36)	Group S (*n* = 18)	Group C (*n* = 18)	$\Delta {{\rm{pain}}}\; {{\rm{scaling}}}\; {{\rm{changes}}}$ of different timepoints (95% CI)	Group *P* value	Time*P* value	Interaction *P* value
WBS					0.000	0.000	0.000
before induction	0	0	0	Reference			
upon wake-up	3.2 (2.5,3.9)	1.6 (0.8,2.3)	4.8 (3.7,5.9)	−3.2 (−4.6,−1.9)^*^			0.000
24 h after surgery	2.6 (1.8,3.4)	1.4 (0.6,2.3)	3.8 (2.4,5.2)	−2.3 (−4.0,−0.7)^*^			0.005
48 h after surgery	1.3 (0.9,1.8)	0.6 (0.1,1.0)	2.1 (1.3,3.0)	−1.6 (−2.5,−0.6)^*^			0.001
FLACC					0.000	0.000	0.000
before induction	0	0	0	Reference			
the time of wake-up	2.5 (1.8,3.1)	0.9 (0.3,1.6)	4.0 (2.9,5.1)	−3.1 (−4.3,−1.8)^*^			0.000
24 h after surgery	2.1 (1.3,2.8)	1.1 (0.3,1.9)	3.0 (1.7,4.3)	−1.9 (−3.4,−0.4)^*^			0.015
48 h after surgery	0.8 (0.4,1.2)	0.3 (0,0.6)	1.2 (0.4,2.0)	−0.4 (−1.7,0)^*^			0.041

∆pain scaling = mean difference of Wong-Baker FACES or FLACC between two groups at the time of wake-up, 24 h after surgery or 48 h after surgery minus mean difference of baseline values before induction between two groups. The covariates adjusted in these models included sex, age, BMI(Body Mass Index) and surgical duration. Data are shown as mean(95% CI).

^*^P < 0.05.

### Hemodynamics results

**Fig. 4:**
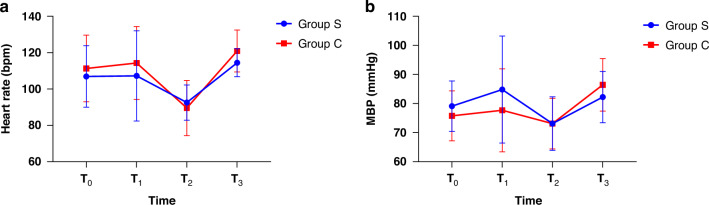
Hemodynamic changes at different time points. **a** HR at different time points and **b** MBP at different time points: T_0_: before induction, T_1_: at the time of intubation, T_2_: at the time of skin incision, T_3_: at the time of extubation. The data are presented as mean ± SD.

There was no significant difference between the two groups over time in HR and MAP at T_0_, T_1_, T_2_, and T_3_. The decrease of HR and MAP was observed in T_2_ compared with the baseline in both groups(*P *< 0.001 and *P *= 0.032). The results of HR and MAP are shown in Table 4, and the trend of HR and MAP of the two groups is displayed in Fig. 4.

**Table 4:** Hemodynamic parameters change according to the GEE model.

	All patients (*n* = 36)	Group S (*n* = 18)	Group C (*n* = 18)	Group *P* value	Time *P* value	Interaction *P* value
HR (bpm)				0.244	0.000	0.194
T_0_	109.1 ± 2.9	106.9 ± 3.9	111.3 ± 4.2			
T_1_	110.8 ± 3.7	107.2 ± 5.7	114.3 ± 4.6		0.967	
T_2_	91.1 ± 2.1^*^	92.6 ± 2.2	89.6 ± 3.5		0.000	
T_3_	117.7 ± 1.6	114.4 ± 1.8	120.9 ± 2.6		0.61	
MAP (mmHg)				0.498	0.000	0.2
T_0_	77.4 ± 1.4	79.1 ± 2.0	75.8 ± 2.0			
T_1_	81.3 ± 2.7	84.8 ± 4.2	77.7 ± 3.3		0.081	
T_2_	73.1 ± 1.5^*^	73.1 ± 2.1	73.1 ± 2.0		0.032	
T_3_	84.3 ± 1.4	82.2 ± 2.0	86.4 ± 2.1		0.231	

The covariates adjusted in these models included sex, age, BMI(Body Mass Index) and surgical duration. Data are shown as mean ± SD.

*HR* heart rate, *MBP* mean blood pressure, *T*_0_ before induction, *T*_1_ at the time of intubation, *T*_2_ skin incision, *T*_3_ extubation.

*P < 0.05.

### Other secondary outcomes and adverse events

The number of cases requiring additional analgesics after surgery in the S-ketamine group was also significantly fewer than in group C at the time of wake-up (*P* = 0.001). Compared with group C, the number of cases 24 h after surgery and 48 h after surgery requiring analgesia in group S was also significantly lower (*P* = 0.003 and *P* = 0.003), as shown in Table 5. There was no significant difference in postoperative nausea and vomiting (PO-NV) and hypoxemia between the two groups (*P* = 0.402 and *P* = 0.177, respectively). There were no cases of hallucination in the two groups. Compared with group S, emergence agitation was significantly increased in group C (*P* = 0.018). The extubation time in each group was 7.3 ± 2.5 min and 8.5 ± 2.0 min, showing no significant difference (*P* = 0.112) (Table 5.)

**Table 5:** Other secondary outcomes and adverse events.

	Group S (*n* = 18)	Group C(*n* = 18)	*P* value
Extubation time, min	7.3 ± 2.5	8.5 ± 2.0	0.112
Rescue analgesia, *n* (%)			
the time of wake-up	1 (5.6%)	10 (55.6%)	0.001^*^
24 h after surgery	5 (27.8%)	14 (77.8%)	0.003^*^
48 h after surgery	5 (27.8%)	14 (77.8%)	0.003^*^
Adverse events, *n* (%)			
Nausea and vomiting,	2 (11.1%)	5 (27.8%)	0.402
Emergency Agitation	1 (5.6%)	8 (44.4%)	0.018^*^
Hypoxemia	1 (5.6%)	5 (27.8%)	0.177

Data are shown as mean ± SD or *n* (%).

### The Spearman rank correlation between infusion time of S-ketamine and postoperative mechanical pain threshold, pain scores in the S-ketamine group

There is a negative correlation between infusion time of S-ketamine and postoperative mechanical pain threshold at 24 h (*r *= −0.570, *P* = 0.014) and 48 h postoperatively (*r *= −0.643, *P* = 0.004). There is no correlation between the infusion time of S-ketamine and pain score at the time of awakening, 24 and 48 h postoperatively. The results of the infusion time and postoperative mechanical pain threshold and pain scores in the S-ketamine group are displayed in Table 6.

**Table 6:** The correlation between infusion time of S-ketamine and postoperative mechanical pain threshold, pain scores in S-ketamine group to the Spearman’s rho.

	Time	Correlation coefficient	*P* value
Mechanical pain threshold	24 h after surgery	−0.570^*^	0.014^*^
	48 h after surgery	−0.643^*^	0.004^*^
WBS	wake-up	−0.249	0.320
	24 h after surgery	−0.128	0.613
	48 h after surgery	0.179	0.476
FLACC	wake-up	−0.309	0.211
	24 h after surgery	−0.048	0.851
	48 h after surgery	0.097	0.703

Time: Infusion time of S-ketamine. Correlation coefficient less than 0 indicates a negative correlation, while correlation coefficient greater than 0 indicates a positive correlation.

*P < 0.05.

The median surgery time is 235 min in S-ketamine group (IQR:285 min). And the median infusion time of S-ketamine is 200 min (IQR: 285 min).

## Discussion

Neoadjuvant chemotherapy for malignant solid tumors in children has become essential to the treatment standard. Chemotherapy considerably advances tumor patients’ survival, but the side effect induced by chemotherapy is pronounced. CIPN persists even after medication is discontinued and beyond the cure of the cancer. Previous studies showed that NMDAR plays a vital role in the etiology and perseverance of chronic (neuropathic) pain. In chronic pain states, the NMDAR is activated and upregulated in the spinal cord (central sensitization), resulting in enhanced signal transmission in the pain circuitry from the spinal cord to the cortex, leading to spontaneous pain, allodynia, and hyperalgesia.^8,21–24^ In addition, the use of remifentanil in the surgery may also lead to secondary hyperalgesia.^25^ This study found that after using S-ketamine, the mechanical pain threshold of patients receiving chemotherapy was significantly higher than that in the control group at 24 h and 48 h after surgery. Previous studies showed that the ability of ketamine may reduce the NMDAR central facilitation, which is consistent with our finding, implying that S-ketamine’s NMDAR antagonism may partly reduce pain sensitivity, thus reversing the pain effect.^16^

In our study, the preoperative Wong-Baker FACES pain rating scale and the FLACC scale of patients were not affected by chemotherapy drugs, showing a 0 score. As previous research reported, the pain in fingers and toes is the most frequent location symptom.^26^ Therefore, there is no feeling of pain at the site of the intended surgical incision in these pediatric patients. However, the Wong-Baker FACES pain rating scale and the FLACC scale at the time of patients’ recovery, 24 h, and 48 h after surgery were significantly higher in group C, indicating that postoperative pain in chemotherapy patients may worsen without effective intervention. Evidence suggests that chemotherapy may promote the expression of proinflammatory cytokines and inflammatory chemokines, which could destroy the homeostasis of the internal environment and exacerbate postoperative pain.^27^ Chemotherapy drugs can also promote the development of postoperative acute pain to chronic pain.^28^ The results of these studies may explain the evolution of postoperative pain aggravation in our research. One research has illustrated that inadequate postoperative pain management is the leading risk factor for chronic post-surgical pain and may increase the risk of adverse events after the surgery.^29^ Therefore, as a significant approach to postoperative analgesia, it is essential to employ effective drug utilization to reduce the incidence of postoperative pain. From our study, in the S-ketamine group, fewer cases need additional analgesics after the surgery, confirming that S-ketamine can reduce the consumption of opioids and alleviate the development of postoperative acute pain and the expression of analgesic activity in chemotherapy patients.^30–32^ Even after the infusion of S-ketamine had stopped, the analgesic effect could continue for more days after surgery. These results were consistent with previous studies’ conclusions of a similar medication of S-ketamine, which demonstrated that ketamine could significantly decrease total pain scores and analgesic use up to 48 h postoperatively.^33,34^

Many studies have demonstrated the reliability and validity of the FLACC scale and the Wong-Baker FACES pain rating scale for pediatric pain assessment, with the FLACC scale being used to determine the observational pain of children aged between 3 and 18, while the Wong-Baker FACES pain rating scale is used as a self-reported scale.^18,19^ However, these scales alone are generally insufficient due to inadequate cognitive development in children under six. Self-report scales like the Wong-Baker FACES pain rating scale depend on the child’s sensory, emotional, and contextual status. So, some younger children have difficulty differentiating simultaneous states (pain or anxiety).^35,36^ A combination of objective and subjective measurement techniques is crucial for successfully assessing children’s pain levels. In this study, the FLACC and Wong-Baker FACES pain rating scales of patients in the S-ketamine group were significantly lower in the control group, increasing the plausibility of the analgesic effects of S-ketamine. On the other hand, the postoperative average score of the Wong-Baker FACES pain rating scale and FLACC scale was closer in the S-ketamine group, which may be related to the fact that S-ketamine used intraoperatively as an analgesic and sedative improved anxiety in children, thereby reducing the impact of pain perception in the postoperative period. There has been a study on ketamine showing similar results.^37^ Previous evidence indicated that patients with chronic pain often have depression or depression-like symptoms, and ketamine has potent antidepressant qualities.^15,38^ It can be inferred that S-ketamine also has a similar effect. This change in emotion may positively impact children’s self-judgments of postoperative pain, which elicited comparability between the Wong-Baker FACES pain rating scale and FLACC scale.

There was no difference between the S-ketamine and the control groups in hemodynamics at different time points. This result may suggest that S-ketamine has little effect on hemodynamic aspects. Mild neurologic side effects were associated with S-ketamine. However, in this study, emergence agitation was not frequent in the S-ketamine group, possibly due to adequate analgesia. In addition, there was no difference between the two groups in discomfort due to PONV, hypoxemia, and dizziness. A meta-analysis also showed that intravenous S-ketamine during the perioperative period did not increase the incidence of PONV.^14^ Still, S-ketamine has been reported to increase the psychotomimetic adverse event rate.^14^ This study reported no hallucinations, possibly related to the psychotic symptom suppression by propofol used during the operation.^39,40^

A subgroup analysis of the S-ketamine group shows a negative correlation between the infusion time of S-ketamine and the postoperative mechanical pain threshold, while there is no direct correlation between the pain score and the infusion time of S-ketamine. The difference may be attributed to the fact that the sensation of pain includes not only sensory but also emotional and cognitive components, while mechanical pain threshold is an objective measurement indicator referring to the minimum mechanical stimulation intensity required to induce pain sensation.^41,42^ Previous studies have also confirmed that pain threshold and pain score reflect the sensitivity of the nervous system and subjective pain experience, respectively. These two parameters may not be entirely consistent.^43^ Ketamine has been shown to reduce central sensitization by blocking NMDA receptors, which may significantly affect pain threshold, but its impact on postoperative pain score may vary among individuals.^44^ This may explain why infusion time in the S-ketamine group correlates differently with mechanical pain threshold and pain score. The change in mechanical pain threshold may reflect the protective effect of medication on the nervous system during surgery, indicating the potential of S-ketamine in preventing postoperative chronic pain or nerve sensitization.^45^ In this study, the infusion time of S-ketamine ranged from 30 to 655 min, suggesting that the beneficial effect can be achieved within this time frame.

### limitations

There were some limitations in the study. First of all, the study had a relatively small sample size, and 10% of participants dropped out, which may have compromised statistical power. However, a subgroup sensitivity analysis had been made, thus assuring the validation of the reliability of the results. A multi-center study should be conducted to confirm the accuracy of findings and enhance recruitment efficiency while improving population diversity and generalizability. Secondly, the administration of a higher or lower dose of S-ketamine may influence the estimation of the safety and effectiveness of postoperative pain. Therefore, further assessment is needed to assess the effect of different doses of S-ketamine for postoperative pain relief in children with preoperative chemotherapy. Thirdly, while this study demonstrated improved postoperative pain score and mechanical pain threshold, the duration of these effects sustained over time is still unclear. Further study should focus on these aspects.

## Conclusion

The intravenous administration of S-ketamine significantly increases the postoperative mechanical pain threshold and reduces the pain level of patients who received neoadjuvant chemotherapy. This effect persists even after the infusion of S-ketamine has stopped, and its impact could last up to 48 h after surgery. Further research is needed to determine the optimal dosage and administration of S-ketamine in chemotherapy patients undergoing surgery. Nonetheless, the results of this study provide promising evidence for the potential benefits of S-ketamine in improving postoperative pain outcomes in this patient population.

The datasets generated during and/or analyzed during the current study are available from the corresponding author on reasonable request.

## Supplementary information

CONSORT-2010-Checklist
